# Demethylation by low-dose 5-aza-2′-deoxycytidine impairs 3D melanoma invasion partially through *miR-199a-3p* expression revealing the role of this miR in melanoma

**DOI:** 10.1186/s13148-018-0600-2

**Published:** 2019-01-16

**Authors:** Cécile Desjobert, Arnaud Carrier, Audrey Delmas, Diego M. Marzese, Antoine Daunay, Florence Busato, Arnaud Pillon, Jörg Tost, Joëlle Riond, Gilles Favre, Chantal Etievant, Paola B. Arimondo

**Affiliations:** 1 FRE no. 3600 CNRS, Epigenetic Targeting of Cancer (ETaC), Toulouse, France; 2 Cancer Research Center of Toulouse, CRCT, Toulouse, France; 3 Department of Translational Molecular Medicine, John Wayne Cancer Institute, Providence Saint John’s Health Center, Santa Monica, CA USA; 4 Laboratory for Functional Genomics, Fondation Jean Dausset – CEPH, Paris, France; 5 Laboratory for Epigenetics and Environment, Centre National de la Recherche en Génomique Humaine, CEA, Evry, France; 6 Institut de Recherche Pierre Fabre, CRDPF, Toulouse, France; 7 UMR 1037 INSERM/Université Toulouse III, CRCT, Toulouse, France; 8 Institut Pasteur CNRS UMR3523, Epigenetic Chemical Biology, Paris, France; 9 CNRS-Pierre Fabre USR3388 ETaC, Toulouse, France

**Keywords:** DNA methylation, microRNA, Melanoma aggressiveness, Epigenetics

## Abstract

**Background:**

Efficient treatments against metastatic melanoma dissemination are still lacking. Here, we report that low-cytotoxic concentrations of 5-aza-2′-deoxycytidine, a DNA demethylating agent, prevent in vitro 3D invasiveness of metastatic melanoma cells and reduce lung metastasis formation in vivo.

**Results:**

We unravelled that this beneficial effect is in part due to *MIR-199A2* re-expression by promoter demethylation. Alone, this miR showed an anti-invasive and anti-metastatic effect. Throughout integration of micro-RNA target prediction databases with transcriptomic analysis after 5-aza-2′-deoxycytidine treatments, we found that *miR-199a-3p* downregulates set of genes significantly involved in invasion/migration processes. In addition, analysis of data from melanoma patients showed a stage- and tissue type-dependent modulation of *MIR-199A2* expression by DNA methylation.

**Conclusions:**

Thus, our data suggest that epigenetic- and/or miR-based therapeutic strategies can be relevant to limit metastatic dissemination of melanoma.

**Electronic supplementary material:**

The online version of this article (10.1186/s13148-018-0600-2) contains supplementary material, which is available to authorized users.

## Background

Cutaneous metastatic melanoma is the deadliest form of skin cancer, with a 5-year survival rate particularly low (< 10%) and increasing incidence [1]. Until recently, patients with metastatic melanoma had few treatment options, including surgery, radiation therapy and/or chemotherapy, giving transient and limited results. Since 2011, new targeted therapies against mutated BRAF (vemurafenib) and immunotherapies such as anti-CTLA4 (ipilimumab) and anti-PD1/PD-L1 antibodies emerged, giving very promising long-term responses. Despite the efficacy of these treatments in clinical trials, resistance rapidly arises after vemurafenib and immunotherapies can induce severe side effects limiting the therapeutic response [2]. In addition, not all patients respond to these therapies and no molecular marker is available to predict patient responsiveness to immunomodulators. Therefore, there is still a strong need for novel strategies.

Interestingly, targeting of the epigenetic regulation is promising for the development of new anticancer treatments in solid tumours [3]. In the past few years, various studies reported on epigenetic alterations in metastatic melanoma, more specifically DNA methylation, and associated them with disease progression [4–9]. DNA methylation, catalysed by DNA methyltransferases (DNMT), is a well-studied epigenetic change in cancer. In the last decade, several epigenetic drugs, such as DNA methylation inhibitors (DNMTi), azacitidine (5aza, Vidaza®) and decitabine (5azadC, Dacogen™), have been approved for the treatment of haematological tumours. In solid tumours, the use of epigenetic therapies is breaking through [10]. Hypomethylation of repetitive DNA elements and hypermethylation of CpG islands located in promoter regions of specific genes are hallmarks of melanoma malignancy [10–13]. Specific methylation signatures were also found for melanoma tumours harbouring BRAF mutations [8, 14]. Thus, the use of an epigenetic strategy, combined with relevant CpG island methylator phenotype (CIMP) [15] as markers for the prediction of prognosis or therapeutic response, could be envisioned for metastatic melanoma treatment [10, 16, 17].

In this context, we investigated the implication of DNA methylation in the aggressiveness of metastatic melanoma and whether the use of demethylating agents can reverse its invasive phenotype. We focused our attention on microRNAs (miRs), since these endogenous non-coding RNAs of 19–25 nt can modulate the expression of several target genes via translational blockade and/or transcript degradation and are broadly involved in cancer progression. Furthermore, some miRs are described to be epigenetically regulated [12, 18] and to participate in the acquisition of invasive capabilities of cancer cells, including melanoma [19–21]. Here, we identified one specific hypermethylated miR gene, *MIR199A2*, whose expression restoration by low doses of 5-aza-2′-deoxycytidine or miR transfection impacts on in vitro cell invasiveness and in vivo metastatic formation.

## Results

### 5azadC impairs melanoma cell invasiveness in a 3D invasion model at low non-cytotoxic doses

**Fig. 1:**
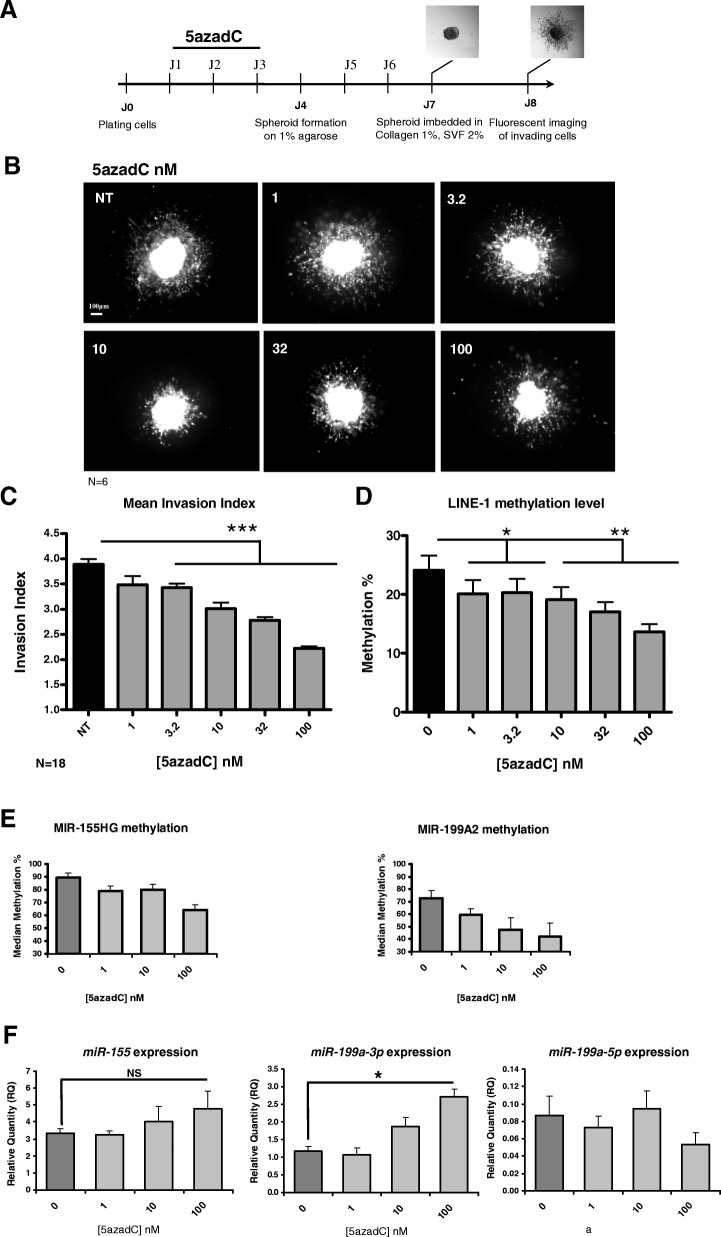
Effect of 5azadC on 3D invasion properties, DNA methylation and specific miR expression in WM-266-4 GFP cells. **a** Experimental scheme for spheroid invasion assays. **b** Six spheroids were analysed for each 5azadC concentration as indicated. Photos shown in **a** are representative of one spheroid per condition. **c** Histogram representing the mean invasion index (invasion area/spheroid initial area) calculated for 18 spheroids. **d** Mean methylation percentage for five CpG sites in LINE-1 sequences. **e** Specific *MIR-155HG* and *MIR-199A2* promoter demethylation was evaluated on spheroids after 5azadC treatment. Median methylation of all the CpG sites is represented. **f** RT-qPCR analysis of mature *miR-155*, *miR-199a-3p* and *miR-199a-5p*. The relative quantity (RQ) was calculated using *RNU6B* gene as control. All experiments were performed in triplicate. SEM are shown and *P value < 0.05, **P value < 0.01, ***P value < 0.001. NS not significant

To date, a limited number of studies have been published depicting the effects of DNMTi on metastatic melanoma aggressiveness and most of them used 2D culture conditions to study invasiveness [22–24], at the exception of the study in combination with IFN-1 [25]. We set up a spheroid invasion assay to explore 3D invasive capacities of metastatic melanoma cells, since this model better preserves cellular features such as cell morphology and cell-cell and cell-extracellular matrix adhesion, known to be important for metastatic dissemination [26]. The metastatic melanoma WM-266-4 cell line constitutively expressing GFP (called herein WM-266-4 GFP) was used to follow the 3D cell invasion. Non-treated and 5azadC-treated WM-266-4-GFP cells were plated to form spheroids, which were embedded in collagen and monitored 24 h afterwards by fluorescent microscopy (Fig. 1a). Prior to measuring its effect on 3D cell invasion, the cell sensitivity to daily treatments of 5azadC, the reference DNMTi, was tested during 3 days. The WM-266-4 GFP cell line displayed a similar viability dose-response curve to the parental cell line WM-266-4, with an EC50 of 100 nM at 7 days (Additional file 1: Figure S2A). In addition, cell sensitivity to 5azadC resulted unchanged under different culture conditions, as shown by comparing 2D and 3D cultured cells (Additional file 1: Figure S2B).

Hence, low concentrations of 5azadC, inducing little cell death and inhibition of cell proliferation (EC50 = 100 nM), were chosen for the 3D invasion assay to limit non-specific cytotoxic effects and favour an epigenetic effect. As illustrated in Fig. 1b, c, 5azadC induced a dose-dependent inhibition of 3D cell invasion, with a significant decrease of the invasion index starting at 3.2 nM (*P* value < 0.001). The anti-metabolic compound cytarabine (araC), structurally similar to 5azadC and commonly used in chemotherapies [27], was used as control at concentrations resulting in < 10% of cell death (1 nM, Additional file 1: Figure S3A). Unlike 5azadC, at the equi-cytotoxic concentration, araC did not cause cell invasion inhibition (Additional file 1: Figure S3B and S3C), confirming a different mechanism of action for 5azadC.

Next, the DNA demethylating activity of the drug was determined by following the global DNA methylation level in spheroids recovered before inclusion in collagen (at day 7, Fig. 1a). The methylation of four CpG sites in LINE-1 elements was chosen as a surrogate marker for global genomic DNA methylation [28]. Figure 1d shows that LINE-1 methylation decreased significantly upon treatment with 5azadC concentrations as low as 1 nM (*P* value < 0.05). This demethylating effect was dose-dependent up to the EC50 measured at day 7 (100 nM).

Altogether, our data revealed that 5azadC displays an anti-invasive effect in a 3D metastatic melanoma model at low concentrations. This effect is correlated to its DNA demethylating action but not to its anti-metabolic and cytotoxic properties, as deduced from lack of effect of araC.

### DNA methylation modulation by 5azadC reactivates *miR-199a-3p*

To shed light on the mechanisms involved in the 5azadC-associated anti-invasive effect, we searched for genes and in particular miRs that were hypermethylated in the metastatic cell line WM-266-4 and which expression could be restored by promoter demethylation. Using data from a genome-wide DNA methylation analysis (BeadChip Illumina 450 K, data not shown), we compared the methylation profile of WM-266-4 (metastatic cells) and its non-invasive counterpart WM-115 cell lines (derived from the primary tumour of the same patient). All genes and miR hypermethylated in WM-266-4 cell line with a difference of at least 20% on three CpGs were selected. Among the 68 hypermethylated miRs, two were chosen for further analysis since they were previously described as playing a role in cell invasion/migration processes: *MIR-155HG* [29] and *MIR-199A2* [30, 31].

The methylation status of CpG sites in the promoter of these two miRs was examined after 5azadC treatment by DNA bisulfite conversion followed by pyrosequencing. For *MIR-155HG*, CpG sites were found hypermethylated in the non-treated WM-266-4 GFP spheroids with a median methylation of 88% (Fig. 1e). Upon 5azadC treatment, the decrease of methylation was comprised between 10 and 23%. For the *MIR-199A2* promoter, CpG sites were found methylated at 73% in non-treated WM-266-4 GFP spheroids and started to be demethylated from 1 nM of 5azadC. A maximum demethylation of 31% was reached at 100 nM (Fig. 1e).

To correlate this DNA demethylation with the respective RNA expression, mature miR levels were monitored in parallel. A dose-response increase of *miR-155* expression was found upon 5azadC treatment without statistical significance (Fig. 1f). Regarding *miR-199a*, two mature miR species are known to be derived from the *miR-199a* precursor: *miR-199a-5p* and *miR-199a-3p*, the former being 13 times less expressed than the latter (Fig. 1f, RQ to RNU6B of 0.086 ± 0.023 vs. 1.16 ± 0.14). A significant RNA re-expression of nearly threefold was found with 100 nM of 5azadC for *miR-199a-3p*, while no significant difference was observed for *miR-199a-5p* (Fig. 1f).

Hence, these results showed that low concentrations of 5azadC lead to DNA demethylation of specific hypermethylated miR promoters in metastatic melanoma WM-266-4 GFP cells. More specifically, *MIR-199A2* promoter demethylation was correlated with the re-expression of its mature form *miR-199a-3p*.

### *MiR-199a-3p* impairs melanoma cell invasiveness and is involved in the anti-invasive effect of 5azadC

**Fig. 2:**
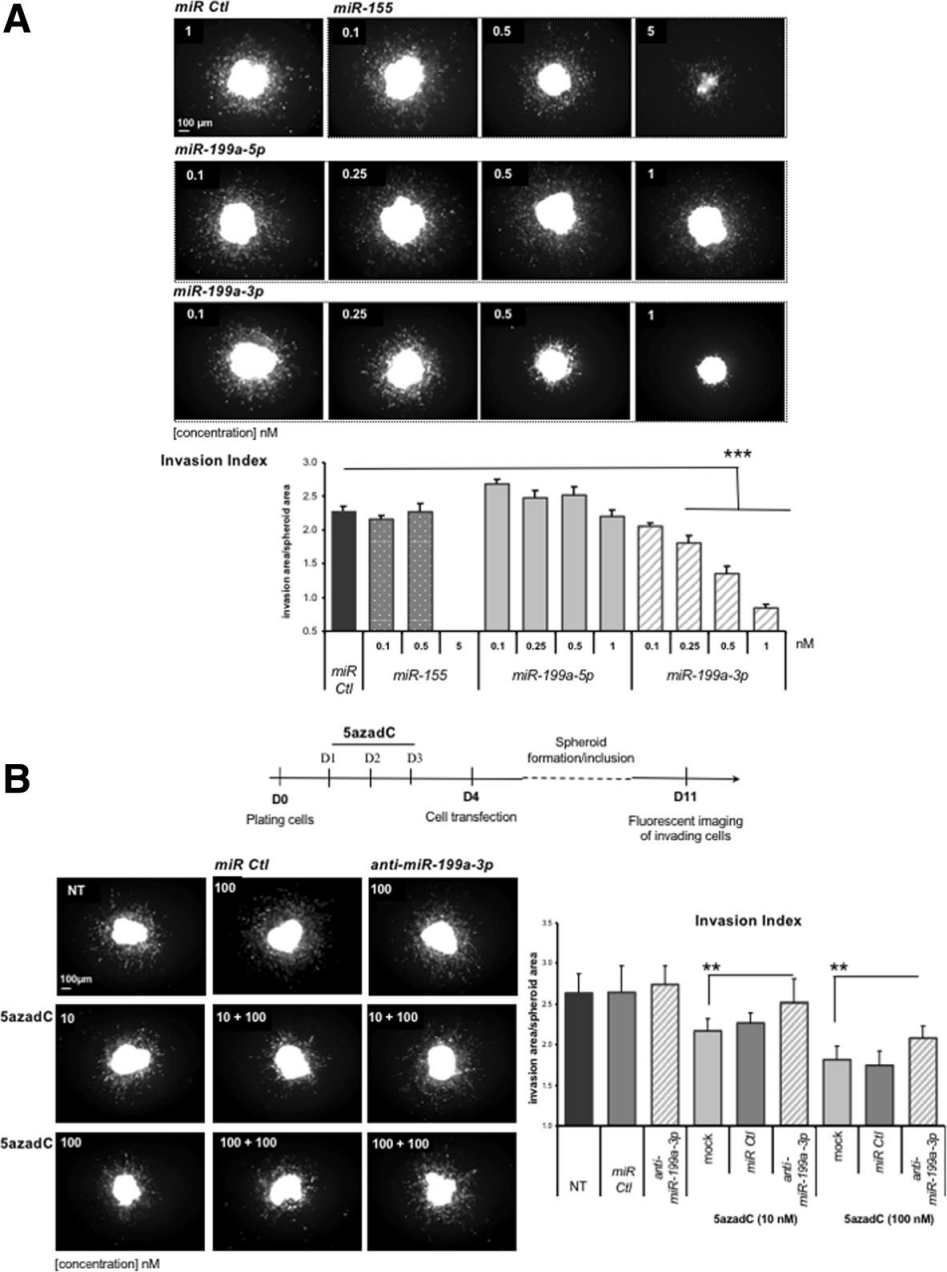
Effect of miR overexpression on 3D metastatic melanoma cell invasion. **a** *MiR-155*, *miR-199a-3p* and *miR-199a-5p* were individually transfected, at the indicated concentration, in WM-266-4 GFP cells 48 h before spheroid formation. Images shown are representative of one spheroid per condition. The mean invasion index and SEM of two independent experiments are shown. **b** Top panel: main experimental steps for 5azadC treatments and *anti-miR-199a-3p* transfection before performing the 3D-invasion assay. Representative images of spheroids for each condition and invasion index quantification are presented. The mean index for 12 spheroids and SEM are shown. *P value < 0.05, **P value < 0.01, ***P value < 0.001, ND not determined, Ctl control and NT not treated

Next, the effect of *miR-199a-3p* re-expression was evaluated in the metastatic melanoma 3D invasion assay. A *miR-199a-3p* mimetic was transiently expressed in WM-266-4 GFP cells 48 h before spheroid formation (Fig. 2). A non-relevant miR (*miR Ctl*), *miR-155* and *miR-199a-5p* were used as controls (Fig. 2a and Additional file 1: Figure S4A). *MiR-155* induced a cytotoxic effect starting from low concentrations (5 nM), as already described by Levati et al. [32]. A drastic reduction of cell invasion was found at very low concentrations of *miR-199a-3p* (Fig. 2a). This anti-invasive effect started from 0.25 nM and reached a maximum at 1 nM (*P* value < 0.001). It was specific to this miR, since its complementary counterpart, *miR-199a-5p*, did not induce such a decrease of the invasion index (Fig. 2a). These observations demonstrated that *miR-199a-3p* alone was able to suppress the 3D invasion ability of WM-266-4 GFP cells, suggesting that its re-expression induced by DNA demethylation can contribute to the anti-invasive effect of 5azadC.

To confirm this hypothesis, *miR-199a-3p* expression was knocked down in 5azadC-treated cells using a specific *anti-miR-199a-3p* (Fig. 2b and Additional file 1: Figure S4B). The 3D anti-invasive ability of 5azadC was significantly reduced, and the invasion index was partially restored (Fig. 2b). These findings support our hypothesis that *miR-199a-3p* plays a role in the 5azadC-mediated anti-invasive effect.

### MET targeting by *miR-199a-3p* is not sufficient to affect 3D cell invasion

**Fig. 3:**
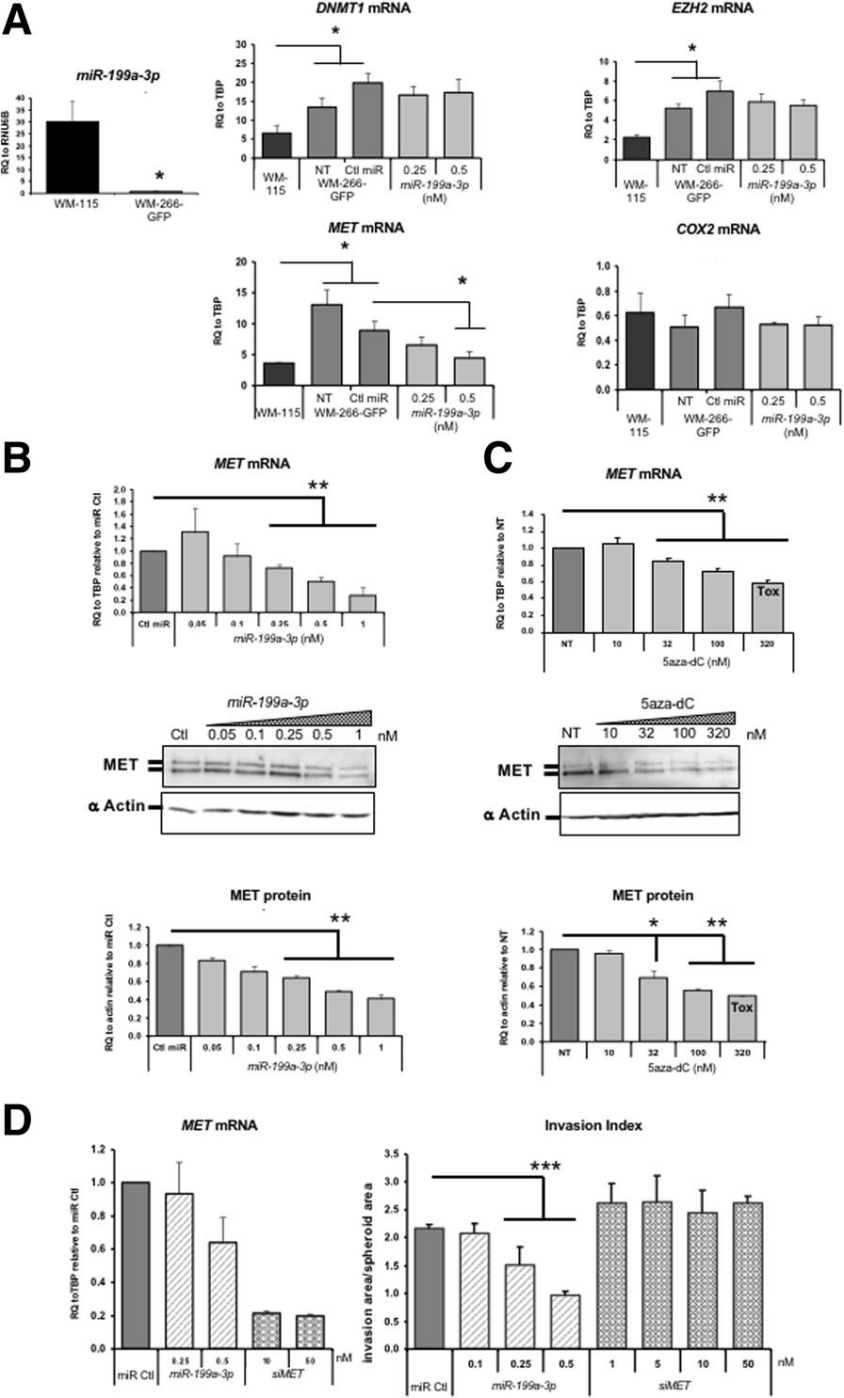
MET as a potential target of *miR-199a-3p* in WM-266-4 cells. **a** Relative expression of *miR-199a-3p*, *DNMT1*, *EZH2*, *MET* and *COX2* in non-metastatic WM-115 and metastatic WM-266-4 GFP cell lines. Results are the mean of quadruplicates. MET mRNA and protein levels were measured upon *miR-199a-3p* ectopic expression (**b**) and 5azadC treatment (**c**). “Tox” indicates more than 50% of cytotoxicity. Histograms for mRNA expression are the mean of at least triplicates normalised to their respective control condition. For Western blots, one representative experiment out of three is shown. Densitometric measurements of MET expression were normalised to actin and relative to the control condition. **d** *MET* mRNA expression was monitored in WM-266-4 GFP cells, transfected with a siRNA targeting MET (siMET) at the indicated concentrations, by RT-qPCR (left panel) before testing cells for 3D invasion (right panel). The mean invasion index is calculated as previously and represented as histograms. SEM are shown. *P value < 0.05, **P value < 0.01, ***P value < 0.001. Ctl control and NT not treated

To better understand the role of *miR-199a-3p* in the invasion process, the combination of bioinformatics and bibliographic approaches was used to identify its target genes. Among the 41 validated mRNAs targets, retrieved in the miRWalk database (http://zmf.umm.uni-heidelberg.de/apps/zmf/mirwalk2/), ten were related to the Gene Ontology (GO) terms of cell motion, cell migration and motility according to DAVID bioinformatics tool [33] (Table S2). In particular, two were already described to be highly expressed in melanomas and involved in the regulation of cell invasion: *PTGS2*, also known as *COX2* [34], and *MET* oncogene [30]. In addition, we chose to study two major epigenetic regulators, *DNMT1* (DNA methyltransferase 1) and *EZH2* (enhancer of zeste homologue 2), since high levels of expression have been associated to melanoma aggressiveness [35]. As shown in Fig. 3a, expression levels of miR-199a-3p was significantly lower in the metastatic WM-266-4 cell line compared to the primary WM-115, while expression of *DNMT1*, *EZH2* and *MET* were significantly higher and no significant differences were observed in the expression of *COX2* mRNA (Fig. 3a). The higher expression of *DNMT1* and *EZH2* is concordant with previous observations in BRAF-mutated cells, such as it is the case for WM266-4, described to participate in the hypermethylated phenotype of metastatic melanoma cells ([36] and personal observations). In our model, *miR-199a-3p* ectopic expression, at concentrations inducing an anti-invasive effect (Fig. 2a), did not affect *DNMT1*, *EZH2* and *COX2* expression (Fig. 3a), but led to the decrease of *MET* mRNA and protein (Fig. 3a, b).

These results suggest an inverse relationship between *miR-199a-3p* and *MET* and thus a possible direct targeting of this oncogene by the miR. A similar decrease of *MET* was also observed upon 5azadC treatment (Fig. 3c), prompting us to hypothesise that re-expression of *miR-199a-3p* following DNA demethylation participates in the downregulation of *MET*. Nevertheless, *MET* knock down was not sufficient to inhibit 3D invasiveness of metastatic melanoma as shown in Fig. 3d. Therefore, *MET* depletion seemed not to be the major event leading to *miR-199a-3p* or 5azadC anti-invasive effect in our model.

### *MiR-199a-3p* and 5azadC deregulate common genes involved in invasion/migration pathways

**Fig. 4:**
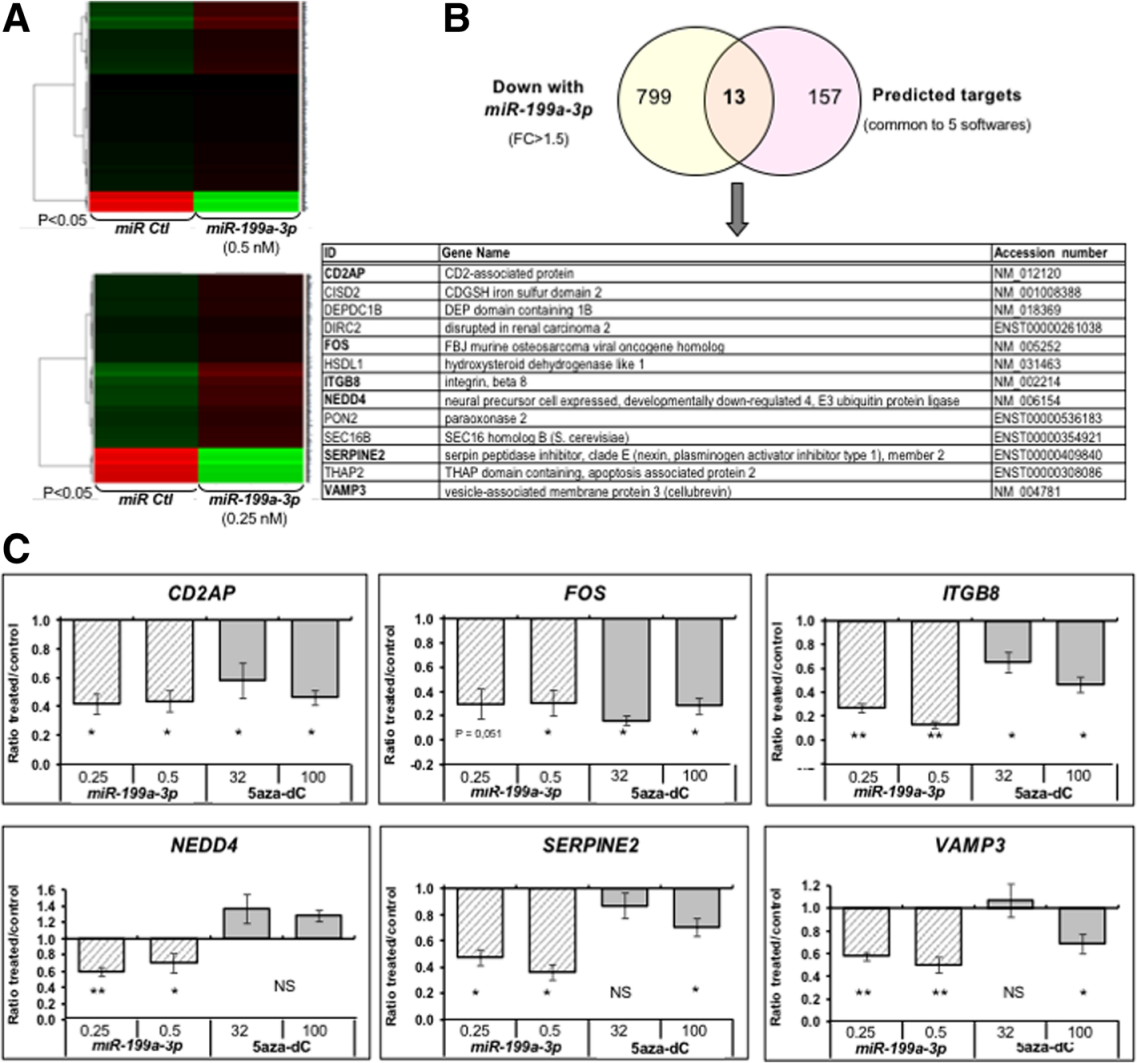
Deregulated pathways and candidate target genes of *miR-199a-3p*. **a** Transcriptomic analysis of WM-266-4 GFP cells transfected with *miR-199a-3p* identified differentially expressed genes with a FC > 1.5. Heatmaps represented all deregulated genes for the two conditions (0.25 and 0.5 nM of *miR-199a-3p*). *P* value cutoff is FDR corrected. **b** Venn-diagram of all downregulated genes with a FC > 1.5 for *miR-199a-3p*-transfected cells (812 genes), crossed with its predicted targets (common to five different softwares). The table summarises the 13 retrieved genes. Genes in bold were further analysed. **c** *CD2AP*, *FOS*, *ITGB8*, *NEDD4*, *SERPINE2* and *VAMP3* expression in WM-266-4 cells transfected with *miR-199a-3p* or treated with 5azadC. The mean fold inhibition was calculated as the ratio of RQ in treated cells/RQ in the control for each gene. SEM are shown for triplicates. *P value < 0.05, **P value < 0.01. NS not significant

To identify other genes targeted by *miR-199a-3p* and involved in invasion processes, we compared global gene expression in *miR-199a-3p* vs control miR transiently transfected cells by transcriptomic microarrays. The differential gene expression analysis, using a fold change threshold > 1.5 and a *P* value < 0.05 (Fig. 4a), revealed 812 downregulated genes.

**Fig. 5:**
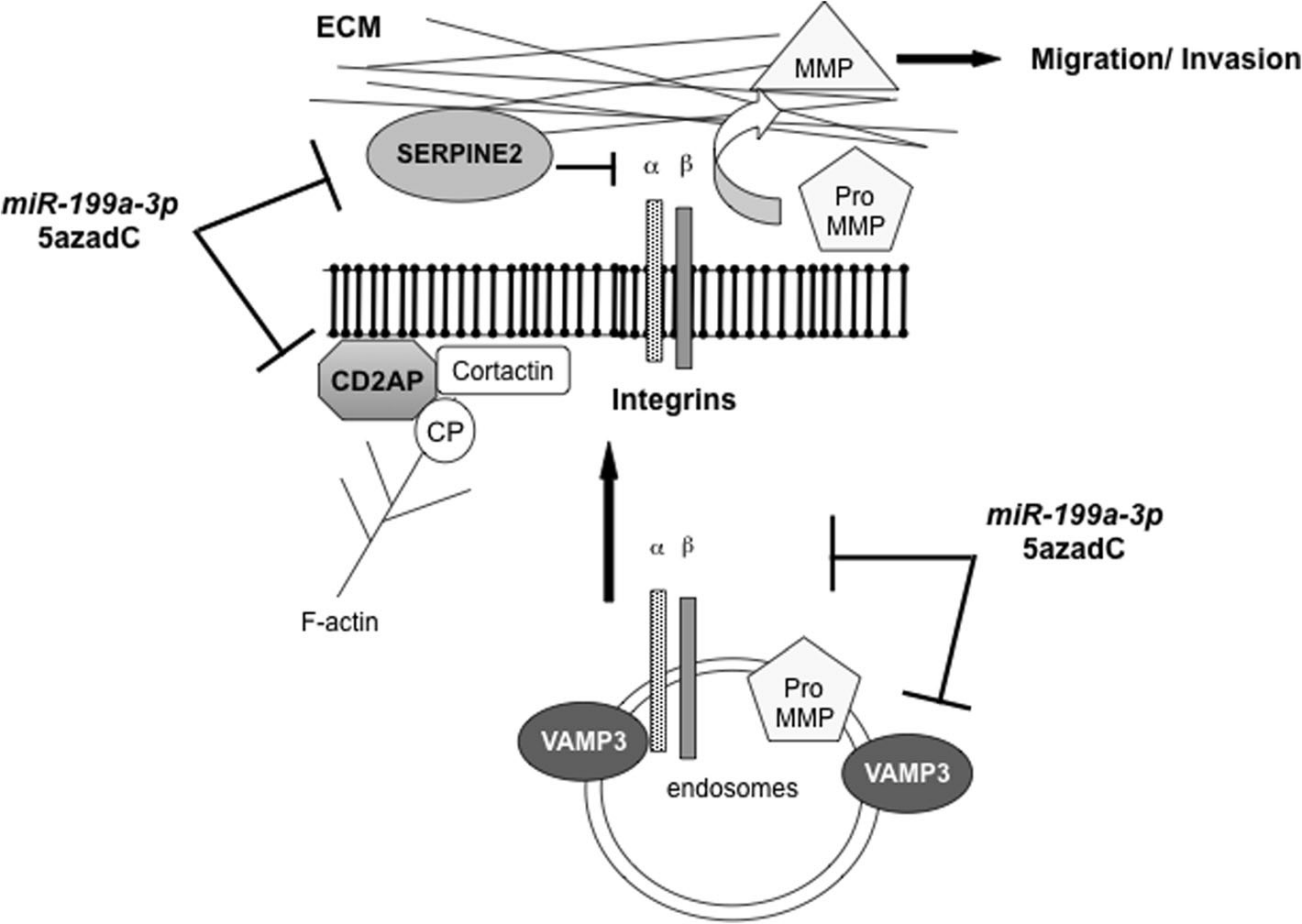
Schematic representation of common downregulated genes by *miR-199a-3p* and 5azadC*.* Genes predicted as targets of *miR-199a-3p* and commonly downregulated by *miR-199a-3p* and 5azadC are highlighted in bold. ECM extracellular matrix, MMP matrix metalloproteinase, VAMP3 vesicle-associated membrane protein 3, CD2AP CD2-associated protein, CP capping protein, SERPINE2 serine protease inhibitor nexin 2

Genes that were downregulated upon *miR-199a-3p* transfection were crossed with gene list of its predicted targets genes by five different softwares (DIANAmT, miRanda, miRDB, miRWalk and TargetScan) on the website: http://zmf.umm.uni-heidelberg.de/apps/zmf/mirwalk2/ (as detailed in the Additional file 1). Among 170, thirteen genes were retrieved (Fig. 4b). Six of them, *CD2AP*, *FOS*, *ITGB8*, *NEDD4*, *SERPINE2* and *VAMP3* (highlighted in bold), belong to cell-signalling or cell migration/adhesion pathways and were chosen for further validation. The ectopic expression of *miR-199a-3p* reduced their expression at the mRNA levels with fold changes ranging from 1.5 to 8 (Fig. 4c). Noteworthy, five out of these genes were also downregulated upon 5azadC treatment (Fig. 4c). CD2AP is a membrane scaffold protein that has a role in dynamic actin assembly and lamellipodia formation. ITGB8, a β-integrin, connects adhesive proteins in the ECM to the intracellular actin cytoskeleton mediating cell adhesion and migration. SERPINE2, a member of the serine protease inhibitor nexin superfamily, contributes to the invasion in solid tumours and more specifically in colorectal cancers bearing KRAS or BRAF mutations [37]. VAMP3, an endosomal SNARE protein, is involved in vesicular trafficking and exocytosis of matrix metalloproteinases MMP2 and MMP9 and β-integrins that are required for cell motility. Finally, FOS forms heterodimers with JUN to reconstitute the transcription regulator AP-1, which participates in skin tumour development by controlling a wide range of cellular processes, including cell migration. Altogether, these results highlighted that 5azadC and *miR-199a-3p* impact on metastatic melanoma invasion properties by downregulating common effectors of this multistep biological process. Figure 5 schematically summarises the possible interplay between the four proteins, CD2AP, ITGB8, SERPINE2 and VAMP3, in the cell migration process, based on the data in the literature.

### Both 5azadC and *miR-199a-3p* alone impair lung metastasis formation by WM-266-4 cells in vivo

**Fig. 6:**
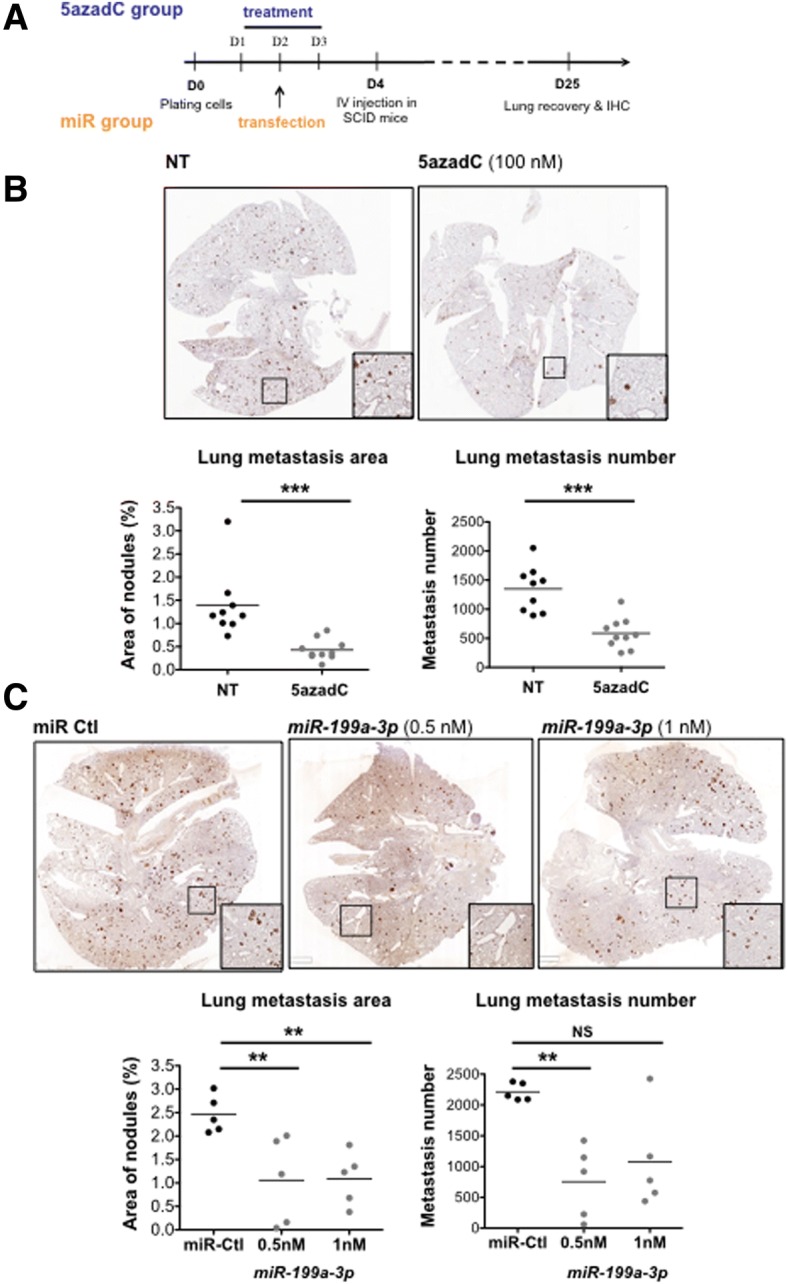
In vivo effect of 5azadC and *miR-199-3p* expression on WM-266-4 lung metastasis*.* **a** Experimental outline: WM-266-4 cells treated or not with 100 nM of 5azadC for 3 days or transfected with *miR-199-3p* mimetic at 0.5 or 1 nM for 48 h were injected in the tail vein (IV) of SCID mice. Lungs were recovered for immunohistochemical analysis 21 days after injection. **b** A representative image of the stained lung is shown for each group. Plots representing the percentage area and the total number of metastasis on one slice for each mouse in non-treated (NT) and 5azadC-treated are shown for two independent experiments. Medians are shown. **c** Same as in B for *miR Ctl* and *miR-199-3p* tested at two concentrations, 0.5 and 1 nM. **P value < 0.01, ***P value < 0.001

To test the anti-tumour effect of 5azadC and *miR-199a-3p*, in vivo experiments were performed using a melanoma model for metastasis formation in the lungs (Fig. 6a). SCID mice were injected in the tail vein with WM-266-4 cells previously treated with 100 nM 5azadC or transfected with 0.5 or 1 nM of *miR-199a-3p*, compared to non-treated or *miR-Ctl*-transfected cells, respectively. Lungs were collected 21 days after the injection, and metastasis numbers and area were counted after tyrosinase staining (Fig. 6a). A significant reduction of both parameters was observed upon 5azadC treatment (Fig. 6b). A similar anti-metastatic effect was observed with *miR-199a-3p*, despite a higher observed heterogeneity (Fig. 6c). These in vivo experiments validate *miR-199a-3p* as an anti-tumour effector and the potential interest of re-expressing this miR or using the demethylating drug 5azadC to limit metastasis formation in melanoma cells presenting a hypermethylated *MIR-199A2*.

### Promoter methylation status and expression of MIR-199A2 in patient samples

**Fig. 7:**
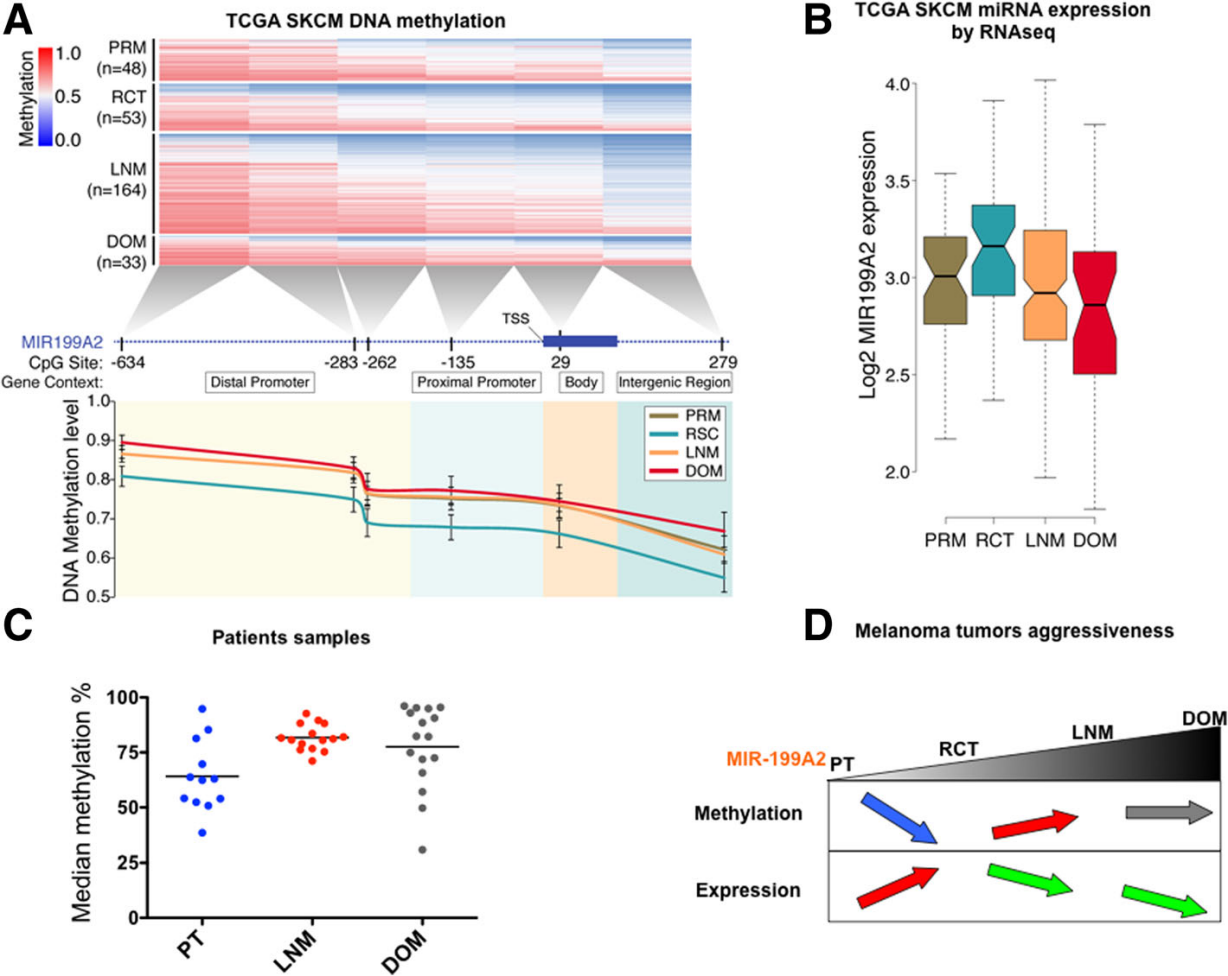
Patient sample analysis and schematic representation of the role of *miR-199-3p* in melanoma aggressiveness. **a** DNA methylation level of six consecutive CpG sites in the genomic vicinity of MIR199A2 gene. TCGA SCKM data was classified according to the melanoma tissue type. **b** microRNA-199A2 expression according to melanoma tissue types. **c** DNA methylation of *Mir-199A2* were analysed in formaldehyde-fixated samples from 12 PT, 15 LN and 16 DM (10 bowel and 6 liver) samples. Median methylation of all the CpG sites is represented. **d** Schematic representation of *MIR199A* promoter methylation and expression as a function of the clinical stages. Blue arrows indicate a decrease in the DNA methylation and red arrows an increase. Green arrows indicate a decrease in the miR expression and red arrows an increase

To further validate the importance of *MIR-199A2* DNA methylation in human melanoma progression, we analysed The Cancer Genome Atlas (TCGA) RNA-seq and DNA methylation datasets available for skin cutaneous melanoma (SKCM) tumours on the UCSC cancer genomics browser website. In Fig. 7a, DNA methylation levels of the *MIR-199A2* promoter were compared according to the tumour site location. This locus was found significantly (*P* value < 0.01) less methylated in the regional cutaneous tissues (RCT) than the primary tumours (PT) of the cohort, while an increase in its methylation was observed in the regional lymph nodes metastases (LNM) in comparison to the RCT (*P* value < 0.05). Consistently, the expression of *miR-199a* was found increased in the RCT, where it was less methylated, and decreased in the LNM and distant organ metastases (DOM), where it was more methylated (*P* value < 0.01; Fig. 7b). We then confirmed these data on a cohort of 43 melanoma patients (Fig. 7c). These observations suggested that this locus is submitted to a tight epigenetic modulation during melanoma progression (Fig. 7d): demethylation at local stages (RCT), methylation and decreased expression at stages that start to disseminate (LN) and maintained at DMs. In summary, these data confirm that *MIR-199A2* is regulated by DNA methylation of its promoter in patients and this methylation varies according to the disease stages. In addition, we observed that the *MIR-199A2* promoter is commonly methylated in cells derived from patients bearing the most aggressive forms of the tumour compared to the primary counterparts with few exceptions (Additional file 1: Figure S5).

Taken together, these data support our hypothesis that *MIR-199A2* hypermethylation can be important at an early stage for the acquisition of an invasive phenotype by the melanoma tumour cells.

## Discussion

Epigenetic therapies are very promising strategies to fight cancer, since epigenetic modifications are reversible and can be chemically modulated. In particular, promoters’ demethylation by 5-azaC or 5-azadC induces the re-expression of several genes or miRs in cancer cells inducing an anti-cancer effect [27]. To date, few clinical trials using epigenetic drugs have been conducted in metastatic melanoma; a recent example is the combination of 5azadC and temozolomide [38, 39]. Yet, epigenetic modifications and, in particular, genomic hypermethylation of CpG islands and miR modulation were shown to sign metastatic melanoma progression [40, 41].

To the best of our knowledge, few reports explored the impact of 5azadC on the cellular mechanism involved in metastatic melanoma, particularly on cell invasiveness, and its potential use as an anti-metastatic drug [23]. To address this question and better mimic the tumour behaviour in vivo, we developed a 3D cell invasion assay using spheroids embedded in a matrix of collagen I, the principal component of the extracellular matrix (ECM). Growing evidence showed that cancer cell spheroids reproduce various traits of micro-tumours, thus providing a better model for assessing tumour invasion [26]. Using the metastatic melanoma WM-266-4 GFP cell line, we showed for the first time that low-cytotoxic concentrations of 5azadC inhibit 3D cell invasion in vitro. In addition, this effect was clearly associated with a global and promoter-specific DNA demethylation. These observations are in complete agreement with Rajaii et al.’s results on uveal melanoma cells, describing that low concentrations of 5azadC reduced both 2D invasion and clonogenicity [23]. Our results in metastatic melanoma cells confirm also the anti-cancer potential of using low doses of the DNA demethylating agents in solid tumours as for haematological cancers [27, 38, 42].

To further elucidate the mechanisms underlying the phenotypical changes induced by 5azadC, we specifically investigated the modifications on miR methylation and expression. MiRs are post-transcriptional regulators that have a broad spectrum of cellular regulation by targeting multiple genes and pathways, including those required for metastatic melanoma progression like cell invasion and migration (reviews [40]). Moreover, their expression or DNA methylation signatures are studied as reliable markers for diagnosis and prognosis of cancer [10]. Here, two miRs, *MIR-155HG* and *MIR-199A2*, implicated in migration and invasion processes, were found hypermethylated in the metastatic melanoma cell line (WM-266-4) compared to its primary counterpart (WM-115). *MiR-155* is an important multi-functional miR involved in the development of multiple solid tumours and is transcriptionally regulated by DNA methylation [29]. However, 5azadC-induced demethylation of *MIR-155HG* promoter (> 20%) was not sufficient to significantly reactivate *miR-155* expression in our metastatic melanoma cell model. Moreover, its ectopic re-expression in the WM-266-4 cells induced a cytotoxic effect rather than an anti-invasive effect. Thus, we focused on *MIR-199A2* that was found hypermethylated in the WM-266-4 cell line and both demethylated and reactivated by 5azadC.

Few reports identified epigenetically modulated miRs in melanomas (reviews [40, 41]). For the first time, we report that *MIR-199A2* locus is under epigenetic regulation in a metastatic melanoma model and it is reactivated by promoter demethylation by 5azadC. In addition, nanomolar concentrations of *miR-199a-3p* impairs 3D cell invasion, unlike *miR-199a-5p*. Furthermore, the anti-invasive effect of 5azadC was counterbalanced by a specific antago-miR suggesting that *miR-199a-3p* takes part of the anti-invasive response induced by 5azadC. Importantly, the transient expression of *miR-199a-3p* in WM-266-4 cells reduced significantly their metastatic potency in vivo, supporting an anti-cancer effect of this miR.

These results are somewhat contradictory to those reported by Pencheva et al. (2012) [43], describing that the cooperation between three miRs, *miR-199a-3p*, *miR-199a-5p* and *miR-1908*, promoted metastasis by impairing two proteins, APOE and DNAJA4, involved in tumour cell invasion and in angiogenesis. This discrepancy can be explained as follows. First, the cellular models used are significantly different. Pencheva et al. selected for their study the most in vivo metastatic subclones of MeWo and A375, two metastatic melanoma cell lines bearing *BRAF* wild-type and *BRAF*V600E mutation, respectively. These subclones overexpressed *miR-199a-3p* and *miR-199a-5p* in different proportions. The A375 subclones, bearing a mutated BRAF, as it is the case of the WM266-4 cell line, overexpressed much higher levels of *miR-199a-5p* (× 10,000) than of *miR-199a-3p* (× 10), strongly suggesting that mainly the *miR-199a-5p* supported the pro-metastatic effect that was observed by the authors in these subclones. In agreement with this hypothesis, we observed a reversion of the anti-invasive effect of *miR-199a-3p* when combined with increasing amounts of the complementary *miR-199a-5p* mimetic (data not shown), revealing a sponge effect of the latter. Second, Pencheva et al. performed in vivo experiments with the combination of the three LNA anti-miRs, targeting *miR-199a-3p*, *miR-199a-5p* and *miR-1908* but not with individual ones or the *miR-199a-3p* mimetic alone. Thus, it cannot be excluded that the balance between each miR level can affect the tumour development. Moreover, cumulative evidences have shown that *miR-199a* has important but versatile functions in tumourigenesis depending on the nature and the stage of the cancer [31]. Indeed, Yang et al. found that low expression levels of *miR-199a-5p* in tumour melanoma tissue samples from patients were associated to the advances of tumour stage [44]. Their observations in B16 melanoma cells and in tumour xenografts seem to indicate that *miR-199a-5p* is a tumour suppressor, via direct targeting of HIF-1α. Third, our findings in metastatic melanoma are in agreement with an increasing number of observations in other cancer types. In testicular tumour, *MIR-199A2* hypermethylation was linked to malignancy progression [45]. In this model, *miR-199a* re-expression led to the suppression of cell growth, cancer migration, invasion and metastasis in vitro and in vivo. Similar observations were made in non-small cell lung cancer, colorectal cancer and breast cancer cell lines [46]. A tumour-suppressive role of this miR was also demonstrated in papillary thyroid carcinoma, in which *miR-199a-3p* was shown to be likely regulated by the BRAFV600E oncogene [47]. Similar anti-cancer functions were described in renal cell carcinoma [48], endometrial carcinoma [49] and prostate cancer [50].

Finally, to better understand the mechanisms involved in the *miR-199a-3p*-mediated anti-invasive effect in metastatic melanoma, its target genes were investigated. The proto-oncogene MET was found overexpressed in the metastatic vs. primary cell line. Despite its downregulation by both *miR-199a-3p* and 5azadC, its specific inhibition was not sufficient to impair 3D metastatic melanoma invasion (Fig. 4). Next, by a transcriptomic and bioinformatic approach, we observed that *miR-199a-3p* targeted several genes involved in key pathways for cell migration/invasion. This alteration of multiple targets can better explain the in vitro anti-invasive and in vivo anti-metastatic effects of the miR. Interestingly, 5azadC also downregulated common effectors of this multistep biological process (Fig. 5). In addition, analysis of TCGA SKCM datasets supports a tight modulation of *MIR-199A2* promoter methylation and *miR-199a* expression during melanoma progression, with local stages (RCT) presenting a promoter demethylation and an increased expression, whereas disseminated tumours (LN) show a *MIR-199A2* hypermethylation concomitant to a decreased repression (Fig. 7). These observations were further confirmed on an experimental cohort of primary melanoma, lymph node and distant metastasis. Altogether, these observations can explain the different effects found in the literature for *miR-199a-3p* depending on its targets and strongly promote the interest of its reactivation at early melanoma stages that start to locally disseminate.

## Conclusions

In conclusion, we proved that slightly cytotoxic doses of 5azadC are translated into an anti-invasive effect in a BRAF-mutated metastatic melanoma model. This effect is in part associated with *MIR-199A2* promoter demethylation and re-expression of its specific mature form *miR-199a-3p*. Indeed, this miR on its own impairs 3D cell invasion by simultaneously targeting multiple genes involved in adhesion/migration processes. This effect was further confirmed in an in vivo xenograft model with transiently transfected cells, thus supporting its implication in metastatic melanoma aggressiveness. Finally, the clinical relevance of our results is confirmed by the analysis of the TCGA cancer skin cohort and 43 patient samples, showing that *MIR-199A2* promoter methylation and expression is finely tuned during melanoma progression. Hypermethylation of its promoter was found associated with LN metastasis stages before distant dissemination (Fig. 7), suggesting its expression extinction could be involved in metastasis progression. Taken together, our findings support the use of epigenetic drugs or miR mimetic-based therapeutic strategies, either alone or in combination, for limiting metastatic dissemination of melanomas. Concordantly, a combination of 5azadC with temozolomide was recently investigated in a phase I/II trial and proven to be safe and improve response and overall survival rates [38].

## Materials and methods

The details of the protocols, including primer sequences, are reported in Additional file 1.

### Human cell lines

The WM-115 and WM-266-4 cell lines were purchased from the European Collection of Authenticated Cell Culture (ECACC), were subjected to cell line authentication via short tandem repeat profiling and were grown up to a maximum of 20 passages and for fewer than 6 months following resuscitation in our laboratory. The WM-266-4 GFP cell line was obtained by transduction with WPXLd, a lentiviral vector encoding GFP, and selected by flow cytometry. Additionally, four primary melanoma cell lines (WC00060, WC00062, WC0008 and WC00081) were purchased from the Coriell Cell Repository (Coriell Institute for Medical Research, Camden, NJ, USA).

### Drug treatments

5-Aza-2′-deoxycytidine (5azadC, decitabine) and cytarabine (araC) from Sigma-Aldrich (St Louis, USA) were dissolved in water at 10 mM and frozen at − 20 °C until use. Serial dilutions were made in culture media and added on cells seeded 24 h earlier at a density of 0.6 × 10^5^ cells/mL in a 12-well plate. Treatment was repeated during 3 days, and cells were harvested at day 4 for further analysis.

### Cell viability assay

The half effective concentration (EC50) of 5azadC or araC was determined at day 7 after the first treatment on WM-266-4 GFP cells using ATP-lite™ assay (Perkin Elmer, France) according to the manufacturer for cells cultured in 2D conditions. This protocol was also adapted for spheroids (3D conditions).

### 3D cell invasion assay

WM-266-4 GFP (3000 cells/well) were allowed to form spheroids for 2 days on agarose 1% (Sigma-Aldrich, #A95–39) coated in 96-well plates, leading to ~ 300-μm-diameter spheroids. Then, six different spheroids for each condition were individually embedded in EMEM media (Lonza, #BE12-684F) containing 1% of Bovine Collagen I (BD, #354231) and 2% SVF. The initial spheroid size (day 7) and the 24-h invasion area (day 8) were measured by fluorescent microscopy, using an Axiovert 200 M device (5X Plan-Neofluor objective, Carl Zeiss, Germany). Fluorescent invasion areas were quantified using ImageJ (NIH) software on the sum of six Z-stacks images (20-μm interval) for each spheroid. Invasion index was obtained by normalising the invasion area at 24 h by the initial spheroid area: invasion area/spheroid area (Additional file 1: Figure S1). Six individual spheroids were quantified for each condition, and three independent experiments were performed.

### Genomic DNA purification

Paraffin-embedded tissue sections were microdissected, and DNA was purified using the ZR FFPE DNA MiniPrep (Zymo Research, Irvine, CA) as reported [51]. Genomic DNA from melanoma cell lines was extracted using DNAzol reagent (Life Technologies, Carlsbad, CA) as in [7].

### Bisulfite modification and pyrosequencing

Quantitative DNA methylation analysis was performed by pyrosequencing of bisulfite-treated DNA as described in [52]. Two PCR were carried out for the five CpGs of interest on the *miR-199a-3p* locus.

PCR1: (1 CpG):

Forward: TTAGGGGTTGTATTTAGTTTTTTTT

Reverse: Biotine-ATTCATTACCAATTCCCCAATCTA

Pyrosequencing primer: GGTTGTGATTTTTAGTTTTGAYGTGGT

PCR2: (4 CpGs):

Forward: Biotine-GTTATTTTGGGGAGGTTTGGGTATG

Reverse: CCCACTTCCTACCCAATTAAAAAAAA

Pyrosequencing primer:

CCATTTTATACACAAACCCATATCTAAAAACAAACRATTCTAACRATCTCTCCAACRACACAACRCATA

### Microarray expression and GO terms analysis

RNA samples were extracted from cells transfected with *miR-199a-3p* miR mimetic as described in Additional file 1. Experiments were performed in three biological replicates for each condition and analysed on Affymetrix Human Gene 2.0 ST arrays. RMA normalisation was applied, and genes differentially expressed between treated and control conditions were determined choosing a threshold of log_2_ fold change at 0.5 and a *P* value < 0.05. *P* value cutoff is FDR corrected [53]. Significantly enriched pathways were chosen using a threshold enrichment score > 2. Data have been submitted to the Gene Expression Omnibus database.

### In vivo metastasis experiments

The animals were handled and cared for in accordance with the Guide for the Care and Use of Laboratory Animals (National Research Council, 1996) and European Directive EEC/86/609, under the supervision of the authorised investigators. Un-anesthetised 7-week-old female SCID mice (Charles River Laboratories, Saint-Germain-sur-l’Arbresle, France) were injected into the tail vein with 3 × 10^6^ viable WM-266-4 cells in 200-μL PBS, previously treated or not with 5azadC at 100 nM (3 × 3 days) or transfected with miR negative control #1 (*miR Ctl*) or *miR-199a-3p* mimic at 0.5 or 1 nM during 48 h. Each group constituted of 4–5 mice. For 5azadC treatment, two independent experiments were performed. Twenty-one days after injection, mice were dissected and lungs recovered, formalin fixed and paraffin embedded. Sections were stained with haematoxylin and eosin (H&E). The number and area of macrometastasis were measured in total lung sections by immunostaining with Tyrosinase antibody Mob299–05 (1/500) (Diagnostic BioSystem, Pleasanton, CA-USA) on an Aperio Scanner (Leica) with Tissue Studio software (Definiens, Munich, Switzerland) for automatic segmentation and quantification of lesions. Statistics were performed using the Mann-Whitney test.

### The Cancer Genome Atlas skin cutaneous melanoma (TCGA-SKCM) dataset analysis

The TCGA-SKCM DNA methylation, RNA-sequencing (RNAseq) and clinical datasets were accessed in June 2016. Wilcoxon-Mann-Whitney test was applied to determine the significance of DNA methylation or gene expression differences between primary and metastatic melanoma tissues.

### Melanoma patients included in the study

Melanoma patients and healthy individuals were included in the study under protocols approved by the Western institutional review board. Informed consent was obtained from all subjects, and the experiments were performed according to the principles set out in the WMA Declaration of Helsinki and the NIH Belmont Report. Tissue specimens were coded according to HIPAA recommendations to ensure the confidentiality of the patients. The specimen cohort included paraffin-embedded tissues including nevi (NEV; *n* = 7), primary melanomas (PRM; *n* = 16), lymph node metastases (LNM; *n* = 15) and distant organ metastases (DOM; *n* = 20). Additionally, cell lines derived from LNM (*n* = 11) were established at JWCI and included in the study. For each patient, median DNA methylation was calculated on five CpGs on the *miR-199a-3p* locus.

## Additional file

Additional file 1: **Table S1.** Target sequences, PCR and sequencing primers used for pyrosequencing assays. **Figure S1.** Measurement of the invasion index. **Figure S2.** Cytotoxic effect of 5azadC on WM-266-4 and WM-266-4 GFP cells. **Figure S3.** Comparison of araC and 5azadC effects on cell viability and 3D invasion. **Figure S4**. *MiRNA* expression levels after transfection. **Figure S5** (A) Mir-199A2 CpG methylation in cell lines. B) RT-qPCR analysis of mature miR-199a-3p in cell lines. (DOCX 1759 kb)

## Abbreviations

5aza: 5-Aza-cytidine
5azadC: 5-Aza-deoxycytidine
araC: Cytarabine
CIMP: CpG island methylator phenotype
Ctl: Control
DNMT: DNA methyltransferase
DNMTi: DNA methyltransferase inhibitors
DOM: Distant organ metastases
EC50: Half effective concentration
LNM: Lymph nodes metastases
miR: MicroRNA
RCT: Regional cutaneous tissues
SKCM: Skin cutaneous melanoma
TCGA: The Cancer Genome Atlas
