# Supplementary material for: Demethylation by low-dose 5-aza-2′-deoxycytidine impairs 3D melanoma invasion partially through miR-199a-3p expression revealing the role of this miR in melanoma
Source: Clin Epigenetics. 2019 Jan 16;11:9. doi: 10.1186/s13148-018-0600-2 (PMC6335767; doi:10.1186/s13148-018-0600-2)
Supplement: Supplementary file 1 — Table S1. Target sequences, PCR and sequencing primers used for pyrosequencing assays. Figure S1. Measurement of the invasion index. Figure S2. Cytotoxic effect of 5azadC on WM-266-4 and WM-266-4 GFP cells. Figure S3. Comparison of araC and 5azadC effects on cell viability and 3D invasion. Figure S4. MiRNA expression levels after transfection. Figure S5 (A) Mir-199A2 CpG methylation in cell lines. B) RT-qPCR analysis of mature miR-199a-3p in cell lines. (DOCX 1759 kb) [file 13148_2018_600_MOESM1_ESM.docx]

**Additional file 1**

| Gene Name | Ensembl Gene ID | Target sequence | Illumina probe ID | PCR Primers on Bisulfite converted DNA | Sequencing Primers |
| --- | --- | --- | --- | --- | --- |
| miR-155HG | ENSG00000234883 | gagaaagggaaaggggaaaacaccaactacc**cg^1^**gg**cg^2^**ctgggcttttt**cg^3^**acttttcctttaaaaagaaaaaagtttttcaagctgtaggttccaagaacaggcaggaggggggagaagggSgggggggttgcagaaaagg**cg^4^**cctggt**cg^5^**gttatgagtcacaagtgagttataaaagggt**cg^6^**ca**cg^7^**tt**cg^8^**cagg**cg^9^cg^10^**ggcttcctgtg**cg^11^cg^12^**GC**CG^13^**AGCC**CG^14^**GGCCCAG**CG^15^**C**CG^16^**CCTGCAGCCTCGGGAAGGGA | **4**-cg17265380  **6**-cg17297071  **12**-cg23433889 | Fwd-MIR155HG_360f20  5'-GAGAAAGGGAAAGGGGAAAA-3'  Rev-MIR155HG_616_9r20  Biotin-5'-TCCCTTCCCRAAACTACAAA-3' | MIR155HG_AMP1_pyro1f14  5'-GGAAAGGGGAAAAT-3'  MIR155HG_AMP1_pyro2f14  5'-GGGGTTGTAGAAAA-3'  MIR155HG_AMP1_pyro3f19  5'-ATAAGTGAGTTATAAAAGG-3' |
| miR-199A2 | ENSG00000208024 | ACTCAAGGGCTGTGATTTCCAGTCTTGA**CG^1^**TGGCACATTTGCTGCAGACTGGGGAACTGGCAATGAATTTAGGACCAGGAAAAGggggagggctgggctggagagtccatatatgggatgatgtcaccctggggaggtttgggtatg**cg^2^**ctgtgc**cg^3^**ctggagagac**cg^4^**ctagaat**cg^5^**cctgctctcagacatgggtctgtgcataaaatggt | **1**-cg24002149  **3**-cg21450888  **5**-cg17178220 | Fwd-MIR199-A2_406f25  5'-ATTTAAGGGTTGTGATTTTTAGTTT-3'  Rev-MIR199-A2_618_9r28  Biotin-5'-ACCATTTTATACACAAACCCATATCTAA-3' | MIR199-A2_AMP1_pyro1f20  5'-GTTGTGATTTTTAGTTTTGA-3'  MIR199-A2_AMP1_pyro2f18  5'-GGGATGATGTTATTTTGG-3' |

Table S1: Target sequences, PCR and sequencing primers used for pyrosequencing assays. Numbers in the sequences indicate the order of CpG analysed by pyrosequencing for each considered gene. Illumina probe ID refers to the CpG sites present on the Infinium Human Methylation 450 BeadChip (Illumina Inc., CA, USA).

**Figure S1**

**Figure S1: Measurement of the invasion index.** The initial spheroid size at day 7 (D7) and invasion area after 24h (D8) shown in red, were measured with ImageJ software on a fluorescent image resulting of the sum of six Z-stacks of 20 µm interval. The invasion index was obtained by dividing the invasion area at 24 h by the initial spheroid size.

**Figure S2**

**
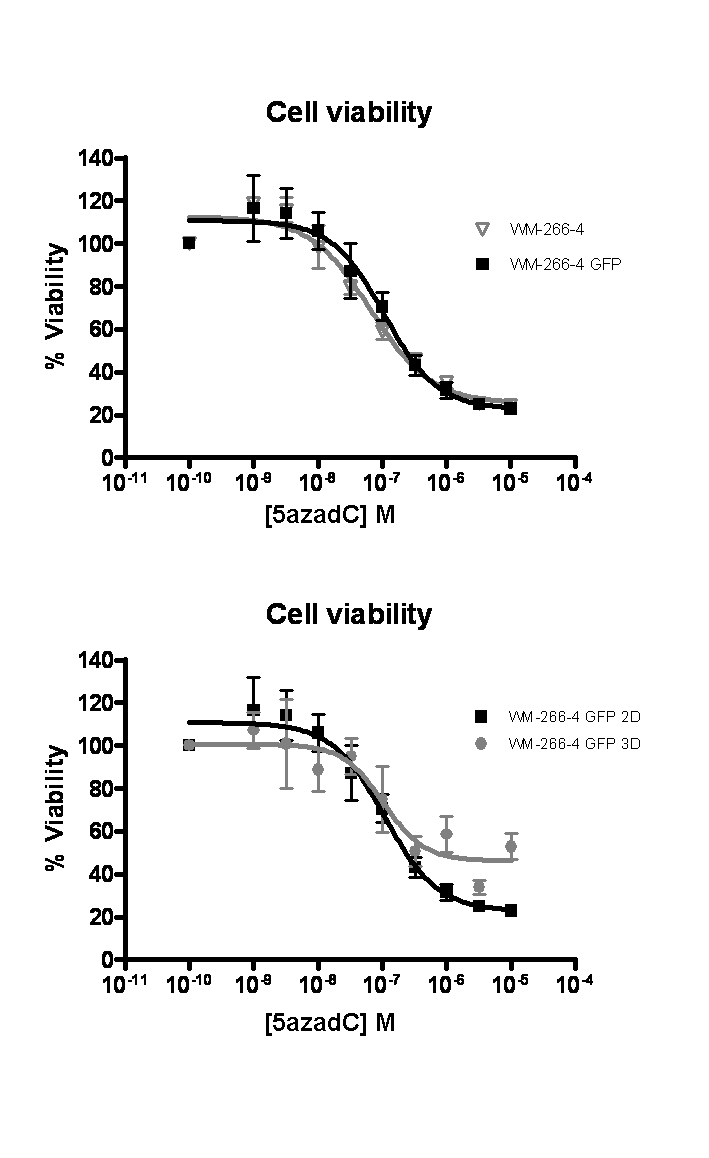
**

**A**

**B**

**Figure S2**: **Cytotoxic effect of 5azadC on WM-266-4 and WM-266-4 GFP cells.** WM-266-4 GFP cell sensitivity to 5azadC was tested upon 2D and 3D culture conditions. An ATPlite™ Luminescence assay was performed on cells at day 7, after three daily treatment of 5azadC at the indicated concentrations. Cell viability curves and EC50 were obtained with GraphPad Prism software. Three independent experiments were performed and SEM are shown.

**Figure S3**


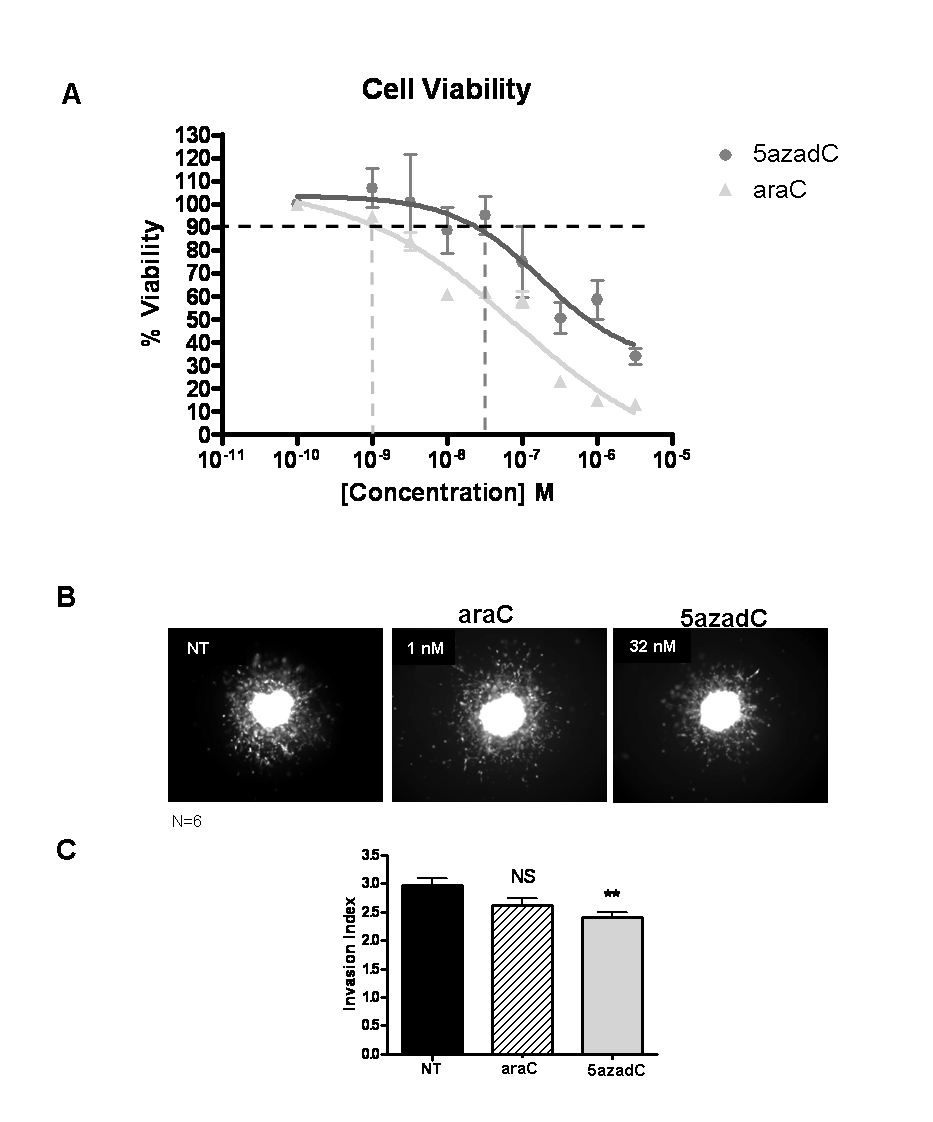


**Figure S3**: **Comparison of araC and 5azadC effects on cell viability and 3D invasion.** (A) Viability curves of WM-266-4 GFP cells cultured in 3D conditions after 5azadC or araC treatments were determined using an ATPlite™ assay. (B) 3D invasion assay was performed at the indicated concentration, corresponding to 10% of cell death. Fluorescent images were taken 24h after spheroid inclusion in collagen I. Photos shown are representative of one spheroid out of six for each condition. (C) Histograms represent the mean Invasion index measured for each compound. SEM are shown. NS: non significant. **: P-value<0.01.

**Figure S4**


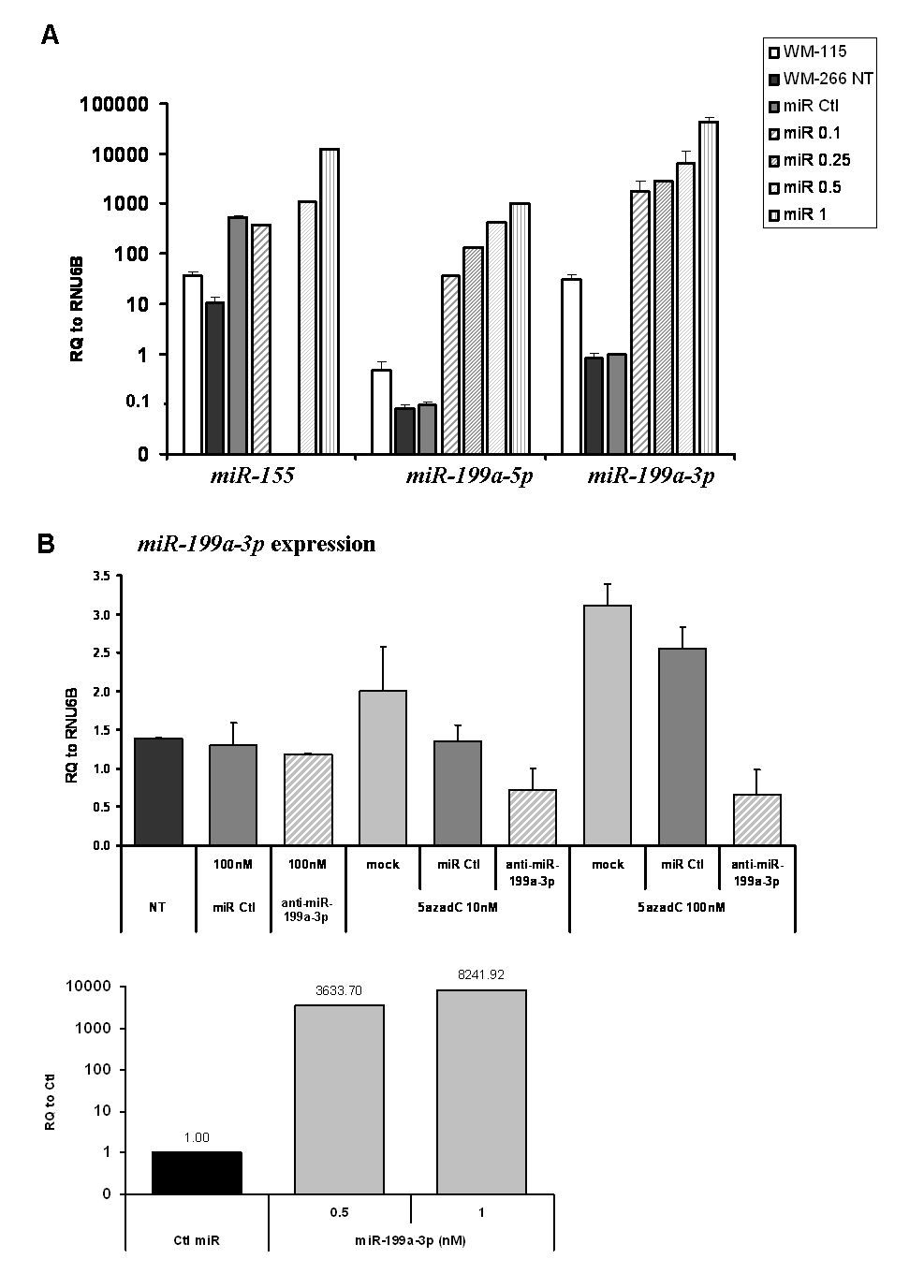


**Figure S4**: ***MiRNA* expression levels after transfection.** (A) RT-qPCR of miRs 48h after WM-266-4 GFP transfection with the indicated concentrations of miRs mimetics (nM). Histograms represent the relative quantity (RQ) to RNU6B. (B) RT-qPCR of miR-199a-3p in 5azadC treated cells. Histograms represent the relative quantity (RQ) to RNU6B. (

**Figure S5**: (A) **Mir-199A2 CpG methylation in cell lines.** The percentage of DNA methylation for each CpG is indicated (each dot corresponds to a CpG). DNA from PT (4) and LN (10) cell lines were established from patient’s melanoma cell culture. CpGs from primary tumours are depicted in blue and in red from metastatic tumours. (B) **RT-qPCR analysis of mature miR-199a-3p in cell lines.** The Relative Quantity (RQ) was calculated using RNU6B gene as control. All experiments were performed in triplicate.

**Supplemental experimental procedures**

**Human cell lines**

The WM-266-4 GFP cell line was obtained by transduction with WPXLd a lentiviral vector encoding GFP: 293T cells were tri-transfected with pWPXLd, pMD2.G and pRSV-Rev kindly provided by Professor Didier Trono. WM-266-4 cells were transduced with the viruses and GFP-expressing cells were selected by flow cytometry. WM239A, WM938A and WM938B cells lines were obtained by ATCC and were subjected to cell line authentication via short tandem repeat profiling and were grown up to a maximum of 20 passages and for fewer than 6 months following resuscitation in our laboratory.

**Cell viability assay**

The half effective concentration (EC50) of 5azadC or araC was determined at day 7 after the first treatment on WM-266-4 GFP cells using cytotoxicity assay according to the manufacturer for cells cultured in 2D conditions (ATP-lite™ assay, Perkin Elmer, France). This protocol was adapted for spheroids (3D conditions) as follows: the spheroids were resuspended in 100 µL of mammalian cell lysis solution and shaked for 20min at 900 rpm. Fifty µl of DMEM, 10% SVF was added to 100 µL of lysed cells transferred in a dark 96 well plate (Greiner, VWR). ATPlite substrate (50 µL) was added and incubated during 15’ at 900 rpm. Luminescent signal was detected on the EnVision 2100 Multilabel Plate Reader (Perkin Elmer) and EC50, which is the concentration where 50% of the maximal effect is observed, was calculated using GraphPad Prism software (GraphPad Software, USA).

**DNA and RNA extractions**

5azadC-treated WM-266-4 GFP cells and spheroids were collected, washed in PBS and dissolved in DNA isolation buffer (0.1 M NaCl, 2% SDS, 50 mM Tris, 10 mM EDTA pH8). Proteinase K (2 mg/mL) was added 2 h at 60°C, followed by RNAse A digestion (40 µg/mL) 30’ at 37°C. The reaction was stopped 10’ at 95°C. Phenol/chloroform/isoamylalcohol extraction was performed adding 2 µL of glycogen (Invitrogen, 20 mg/mL) and the DNA was precipitated with 2xVolums of ethanol 100% and 1/10^th^ NaCl 2 M, overnight at -20°C. The DNA pellet was washed with 70% ethanol, dried at room temperature and dissolved in 20 µL of molecular biology grade water.

Total RNA extraction was performed on 25 to 40 spheroids or on 500.000 cells, using Tri Reagent (Sigma-Aldrich) for total RNA isolation, following the manufacturer’s protocol and adding glycogen. RNA were resuspended in 20 µL RNase free water. DNA and RNA concentrations were measured on a NanoDrop 2000 Spectrophotometer (Thermo Fisher Scientific, Germany). RNA quality was controlled on a Agilent 2 100 Bioanalyser using the RNA nano assay and processed at the “GeT-biopuces” Research Facility LISBP (Toulouse, France).

**Bisulfite modification and pyrosequencing**

Quantitative DNA methylation analysis was performed by pyrosequencing of bisulfite-treated DNA as described in ([Tost and Gut, 2007](#_ENREF_88)). For LINE-1 repeated sequences, the following primers were used : forward primer (5’-TTTTGAGTTAGGTGTGGGATATA-3’) and reverse primer (Biotin-5’-TCTCACTAAAAAATACCAAACAA-3’) for the PCR and (5’-GGGTGGGAGTGAT-3’) for the pyrosequencing primer. The CpG sites in promoters of MIR-155HG and MIR-199A2 genes are described in the supplementary Table S1. The methylation status of these respective CpG sites was analysed on the genomic DNA by bisulfite conversion followed by PCR and DNA pyrosequencing using primers described in Table S1.

**miRNA and siRNA cell transfections**

MirVana™ miRNA mimics, miRNA inhibitors and the miRNA negative control #1 (#4464058) were purchased from Life Technologies. The Human MET siRNA ON-Target plus Smart pool was purchased from Thermo Fisher Scientific (#L-003156-00-0005). The lyophilised powders were resuspended at 50 µM in RNase free water. Exponentially growing cells were seeded at 160.000 cells/500 µL in 24-well plates and transfected with miR mimics, miR inhibitors or siRNA MET at the mentioned concentrations and using Lipofectamine^TM^ 2000 Reagent (Life Technologies) according to manufacturer’s instructions. Cells were recovered 48 h after transfection for further analysis.

**mRNA and miR expression analysis**

For mRNA expression, RT-qPCR was performed on total RNA extracted as mentioned above. Total RNA (500 ng) was reverse transcribed into cDNA with the iScript cDNA Synthesis Kit (BioRad, Marne-La-Coquette, France). Real time PCR was performed with 1/10 dilution of RT product using SsoAdvanced universal SYBR Green supermix from BioRad and RT^2^ pPCR primers (Qiagen, Germany) at 350 nM for each gene of interest (*CD2AP* #PPH18239A, *FOS* #PPH00094A, *ITGB8* #PPH00647A, *MET* #PPH00194A, *NEDD4* #PPH7084B, *PTGS2* #PPH01136F, *SERPINE2* #PPH08354A, *VAMP3* #PPH06289A) on a CFX384 Touch ™ Thermal Cycler (BioRad). PCR program was as follow: an initial denaturation step of 95°C for 2’, 40 cycles of 95°C 10’’, 60°C 30’’, ended by a fusion cycle. Data were analysed with the CFX Manager v3.0 software to generate the Ct values and calculate the relative expression by the 2^-ΔCt^ method using TBP as the control gene.

For mature miRNAs detection, cDNA were synthesized from total RNA using gene-specific primers for *hsa-miR-155, hsa-miR-199a-5p* and *hsa-miR-199a-3p*, according to the TaqMan MicroRNA assay protocol (Life Technologies). Real-time PCR for each individual miRNAs was performed using a Bio-Rad CFX384 (BioRad) detection system. Normalization of target miRNAs was done with the *RNU6B* small RNA*.* Data were presented as relative quantity (RQ) of target miRNA and calculated by the 2^-ΔCt^ method.

**GO terms analysis**

Genes that were down-regulated upon *miR-199a-3p* transfection were crossed with genes list of its predicted targets by five different softwares (DIANAmT, miRanda, miRDB, miRWalk and TargetScan) on the web site: <http://www.umm.uni-heidelberg.de/apps/zmf/mirwalk/>. DAVID bioinformatics resource (<http://david.abcc.ncifcrf.gov/home.jsp> (Huang *et al.* 2009, Huand da *et al.* 2009) was used for GO terms (Biological Process, Molecular Function and Cellular Component) classification. Significantly enriched pathways were chosen using a threshold enrichment score >2.

**Western blotting**

The cell pellet was resuspended in Super RIPA buffer containing protease inhibitor cocktail (Sigma-Aldrich) and phosphatase inhibitors (NaF 5mM, Na3VO4 1mM). Protein lysates were separated using 8% SDS-acrylamide gels, and transferred onto PVDF membranes (Whatman, GE Healthcare, UK). For immune-detection, membranes were incubated with antibodies directed against MET at 1/1000 (D1C2, Cell signalling, USA), or α-actin at 1/10000 (MAB1501, Merck Millipore, Germany) in TBS-Tween 0.1% and milk 5%. Signals from HRP coupled secondary antibodies were generated by enhanced chemiluminescence solution (Immobilon, Merck Millipore) and recorded with G:BOX Chemi XR5 imaging system (Syngene, UK). Densitometric quantification was performed using GeneTools software from Syngene. Experiments were repeated in triplicate.

**The Cancer Genome Atlas Skin Cutaneous Melanoma (TCGA-SKCM) dataset analysis**

Samples were segregated according to their site localization into Primary tumours (PT), Regional cutaneous tissue (RCT), Regional lymph node (LN) and Distant metastasis tumours (DM).

**Bibliography**

Tost, J., and Gut, I. G. (2007). DNA methylation analysis by pyrosequencing. Nat Protoc *2*, 2265-2275.

Huang DW, Sherman BT, Lempicki RA (2009). Systematic and integrative analysis of large gene lists using DAVID Bioinformatics Resources. Nat Protoc 4, 44-57.

Huang da W, Sherman BT, Zheng X, Yang J, Imamichi T, Stephens R, Lempicki RA (2009). Extracting biological meaning from large gene lists with DAVID. Curr Protoc Bioinformatics. 2009 *37*, 1-13.
